# Job satisfaction and motivation among public sector health workers: evidence from Ethiopia

**DOI:** 10.1186/s12960-015-0083-6

**Published:** 2015-10-29

**Authors:** David R. Hotchkiss, Hailom Banteyerga, Manisha Tharaney

**Affiliations:** Department of Global Community Health and Behavioral Sciences, School of Public Health and Tropical Medicine, Tulane University, New Orleans, Louisiana USA; Addis Ababa University and Miz-Hasab Research Center, Addis Ababa, Ethiopia; FHI 360, Washington DC, USA

## Abstract

**Background:**

Although human resources for health have received increased attention by health systems decision-makers and researchers in recent years, insufficient attention has been paid to understanding the factors that influence the performance of health workers. This empirical study investigates the factors that are associated with health worker motivation over time among public sector primary health care workers in Ethiopia.

**Methods:**

The study is based on data from public sector health worker surveys collected through a convenience sample of 43 primary health care facilities in four regions (Addis Ababa, Oromia, Amhara, and Somali) at three points in time: 2003/04, 2006, and 2009. Using a Likert scale, respondents were asked to respond to statements regarding job satisfaction, pride in work, satisfaction with financial rewards, self-efficacy, satisfaction with facility resources, and self-perceived conscientiousness. Inter-reliability of each construct was assessed using Cronbach’s alpha, and indices of motivational determinants and outcomes were calculated for each survey round. To explore the associations between motivational determinants and outcomes, bivariate and multivariate regression analyses were carried out based on a pooled dataset.

**Results:**

Among the sample public sector health workers, several dimensions of health worker motivation significantly *increased* over the study period, including two indicators of motivational outcomes—overall job satisfaction and self-perceived conscientiousness—and two indicators of motivational determinants—pride and self-efficacy. However, two other dimensions of motivation—satisfaction with financial rewards and satisfaction with facility resources—significantly *decreased*. The multivariate analyses found that the constructs of pride, self-efficacy, satisfaction with financial rewards, and satisfaction with facility resources were significantly associated with the motivational outcomes, after controlling for other factors.

**Conclusions:**

Overall, the findings support the premise that both financial and non-financial factors are important determinants of health worker motivation in the Ethiopian context. Although the findings do not point to specific interventions that should be introduced, they do suggest possible areas that interventions should target to help improve health worker motivation.

**Electronic supplementary material:**

The online version of this article (doi:10.1186/s12960-015-0083-6) contains supplementary material, which is available to authorized users.

## Background

Health workers account for the largest share of public expenditures on health and play a crucial role in efforts to improve the availability and quality of health services. However, there is concern that poor health worker performance may be limiting the effectiveness of health systems strengthening efforts [1]. A critical factor affecting health worker performance is worker motivation, which has been defined as the individual’s degree of willingness to exert and maintain an effort towards organizational goals [1,2]. Although it cannot be directly observed, worker motivation has been described as an internal psychological process and a transactional process that results from the interactions between individuals, the organizations for which they work, and the broader societal context [2]. Because high motivation can lead to better performance and high levels of satisfaction among workers, a better understanding of health worker motivation is essential to design effective health care delivery systems. However, despite the importance of understanding health worker motivation, relatively little empirical evidence is available on this issue from low- and middle-income countries [1,3,4].

In Ethiopia, there is concern that low health worker motivation may be undermining the success of health sector reforms that the government has introduced over the past decade as well as disease-focused health programs, including those supported by global health initiatives such as The Global Fund to Fight AIDS, Tuberculosis and Malaria and the United States President’s Emergency Plan for AIDS Relief (PEPFAR). Specific initiatives that have been introduced by the government include the following: the Health Extension Program (HEP), which has trained and deployed health extension workers in communities; efforts to improve aid effectiveness, such as the International Health Partnership; district-based training, which aims to better meet local needs; expansion of pre-service training in order to increase the physician and nurse workforce; and expansion and upgrading of health care facilities in order to increase access to health care services [5]. Previous researchers have found that health workers in Ethiopia tend to be unsatisfied with many aspects of their job, especially their salary, their training opportunities, and their chances of promotion [6,7]. However, there have not been any previous studies in Ethiopia that explore the determinants and consequences of health worker motivation nor changes over time.

The purpose of this study is to empirically assess the determinants and outcomes of health worker motivation over time among public sector health workers in Ethiopia. The study is based on health worker surveys conducted using a standardized instrument to identify key work factors related to motivation and motivational outcomes. The surveys were carried out in a purposive sample of 57 primary health care facilities in four regions (Addis Ababa, Oromia, Amhara, and Somali) at three points in time—2003/04, 2006, and 2009—as part of a separate study that used a mixed-methods approach to investigate the health-system-wide effects of global health initiatives that have supported Ethiopia’s HIV/AIDS, tuberculosis, and malaria programs [8]. It is hoped that the results presented in this article can be useful to policymakers responsible for improving strategies designed to improve health worker motivation and performance and, in turn, health system functioning and population-based health outcomes.

## Methods

### Conceptual framework

Previous theoretical and empirical research suggests that health worker motivation is a complex process involving many layers of influences, including determinants at the individual, organizational, and societal levels [2,3]. Both financial and non-financial factors can influence health worker motivation. A number of studies have found that the perceived insufficiency of salary levels partly explains low levels of motivation [9-11]. In addition, non-financial factors have been found to play a large role in determining health worker motivation. Such factors include resource availability, opportunities for training and promotion, supervision, and management and communication within the organization [2,3,9,12,13].

**Figure 1:**
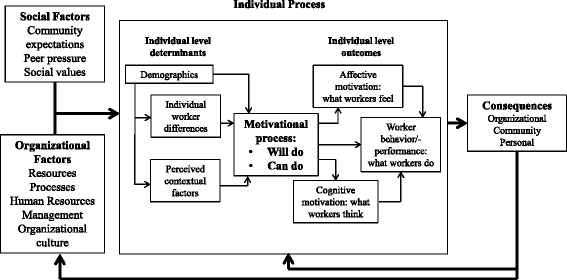
**Conceptual framework of the determinants and consequences of health worker motivation.**

The approach for the study draws on the conceptual framework of the determinants of health worker motivation proposed by Franco et al. [2] based on research carried out in developed and developing countries. As depicted in Figure 1, health worker motivation is viewed as a dynamic psychological process that results from the transaction between individuals and their work environment. Motivation is determined by the congruence of worker and organizational goals (“will do” motivation) and factors that are focused on goal striving (“can do” motivation). “Will do” motivation is influenced by (a) distal determinants such as societal and cultural values, personal values, and personality tendencies and (b) proximal determinants that are more amenable to policy change, such as organizational structure and culture, management practices, financial rewards, and non-financial recognition. “Can do” motivation refers to factors that influence goal accomplishment following goal adoption, such as self-concept, work orientation, self-confidence, and self-regulatory skills. The outcomes of motivation consist of three domains: behavior (job performance), affect (health worker satisfaction), and cognitive aspects (work attachment) of health workers.

### Survey data and sampling

Data for the study come from health worker surveys administered within a panel of primary health care facilities in four regions in Ethiopia—Addis Ababa, Oromia, Amhara, and Somali—at three points in time: 2003/04, 2006, and 2009. As mentioned in the introduction, the data were collected as part of a larger study that investigated the system-wide effects of global health initiatives [8]. The surveys were administered by the Miz-Hasab Research Center, a research organization based in Addis Ababa, Ethiopia. Three of the four regions were selected for the study because (1) they are large and densely populated; (2) they have large urban settlements with access to road transportation; (3) they had at the time of the baseline survey high prevalence rates of HIV/AIDS/STI, TB, and malaria; and (4) HCT, PMTCT, and ART activities were provided in these regions. Somali region was selected because (1) it is remote, (2) it has pastoral communities, and (3) it had a high malaria prevalence level at the time of the selection.

All types of primary health care units (health posts, health stations/clinics, and health centers; public, private for-profit, and NGO entities) were included in the survey. The number of facilities selected in each region was approximately proportional to the total number of health facilities within that region, by type of facility. Facilities were chosen in consultation with regional health officials so as to minimize travel time while ensuring adequate representation of rural and urban areas.

Of the 60 primary health care facilities surveyed in 2003/04, 21 were government health centers, 24 were government clinics and health posts, and the remaining 15 were private health facilities, including both for-profit and non-governmental organization facilities. An attempt was made to revisit the same facilities for the subsequent two surveys conducted in 2006 and 2009. Of the 60 facilities visited in 2003/04, 57 were revisited for rounds two and three (43 public sector facilities and 14 private health facilities). Among the public sector facilities, some health posts and health clinics were upgraded to health centers between survey rounds as part of the government’s strategy to scale up the availability of health care services.

All health workers present at the time of the visit to the facility were surveyed. No attempt was made to return to the facility to interview those who were not present. In addition, because of worker turnover and anonymity requirements, no attempt was made to track the same health workers over time.

Due to the relatively small number of private sector facilities included in the sample (14 of the 57 facilities that were visited in all three survey years), we chose to restrict the sample for this study to public sector health workers. The number of public sector workers surveyed was 234 in 2003/4, 246 in 2009, and 312 in 2009, yielding a total sample size of 792 for the pooled dataset.

### Data collection instrument

The health worker survey included questions on type of position, in-service training, work experience, supervision, workloads^1^, perceptions of resource availability, whether the respondent is proud to work at the facility, self-efficacy, and self-perceived conscientiousness, overall job satisfaction, and satisfaction with financial remuneration.

The questionnaire was based on an instrument developed by Bennett et al. [14] to investigate health worker motivation in Georgia and Jordan. However, only a subset of the questions on health worker motivation was included for this study, which means that we did not have data to measure the full set of motivational determinants and consequences included in the conceptual framework. The research team that developed the initial research design vetted the questionnaires with a technical steering committee that was established for the study. The committee included senior officials from The Global Fund for HIV/AIDS, TB, and Malaria’s Country Coordinating Mechanism for Ethiopia, the Federal Ministry of Health, and the World Health Organization. The instrument was pre-tested in Ethiopia and revised based on pre-test results.

Following our conceptual framework, perceptions of resource availability and financial rewards are viewed as organizational characteristics, pride is viewed as a measure of organizational culture, and self-efficacy is viewed as a measure of individual work-related personality. The consequences of worker motivation are measured with indicators of job satisfaction and self-perceived consciousness. Data on the performance of individual health workers were either not available or were considered to be of insufficient quality to be included in the study (i.e., hours worked).In collecting data on the potential determinants and outcomes of health worker motivation, sample workers were asked to respond to a number of statements classified on a Likert scale of 1–5, with “1” signifying strong disagreement with the statement and “5” representing strong agreement with the statement. The statements were shaped around five different components of health worker motivation: the amount of pride they take in their work, their satisfaction with the financial compensation they receive, their perceived self-efficacy in being able to do their job, the availability of resources to assist them with performing their job adequately, and their self-perceived conscientiousness. For each of these constructs, Cronbach’s alpha was used to examine the inter-reliability of the scales and most of the scales had acceptable alpha levels (Additional file 1: Table S1).

### Measures

Several indices were constructed for the study. Each of the indices was created as follows. First, statement-specific variables were created, which take on the value of 1.0 if the worker strongly agrees with the statement, 0.5 if the worker agrees with the statement, and 0.0 if the worker either has no opinion, disagrees, or strongly disagrees. The statement-specific indicators were aggregated to obtain a raw score, and then, the raw score was converted into a percentile score. The specific measures used in the study are described below.

### Resource availability

To investigate perceptions of workforce availability, an index was created to summarize health worker responses to statements regarding the resources available at their health facility. Topics included the following: whether the lack of equipment and supplies prevented them from doing their job well; whether they had the necessary supplies, equipment, and resources to do a good job; whether the health facility provided everything they needed to do their job well; and whether the lack of supplies hindered the quality of services delivered.

### Financial rewards

This motivation index summarizes the health worker’s satisfaction with his or her financial remuneration. It is based on whether the worker strongly agrees or agrees with statements on whether the effort the workers put in at the facility is reflected in their pay; whether the job pays adequately compared to other jobs; whether the income was a fair reflection of the person’s skill, knowledge, and training; and whether the wage covers their basic needs and is enough to support their family.

### Pride

This motivation index measures the intensity of the health worker’s pride in working at the health care facility. It is based on whether the worker strongly agrees or agrees with the following statements: the facility has a good reputation in the community, it was a source of pride to get a job at this facility, the majority of the workers in this facility are proud to work here, and the workers pride themselves on providing good services to the patients.

### Perceived self-efficacy

This motivation index summarizes health workers’ level of agreement with statements on their confidence to handle the work and to cope with any new challenges that occurred in their work life, their confidence that things were going the way they wanted them to, that they had control over things concerning their work, and that they had received sufficient training to perform well.

### Job characteristics

To measure the motivation properties of the job, respondents were asked “How satisfied are you with your chances to accomplish something worthwhile?”, “How satisfied are you with the chances you have to learn new things”, and “How satisfied are you with the chances you have to do something that makes you feel good about yourself as a person?”. For each question, respondents were asked to choose a number that best describes their level of satisfaction, with “1” indicating “not at all satisfied” and “5” indicating “very satisfied.”

Two indicators of motivational outcomes were measured: job satisfaction and conscientiousness, as assessed by respondents.

### Job satisfaction

To investigate job satisfaction, respondents were asked “all in all, how satisfied are you with your job?” using the Likert scale describe above.

### Self-perceived conscientiousness

This index measures health workers’ level of agreement with statements concerning their own reliability and dependability at work. Statements in this category concerned the ability of the health worker to be reliable and dependable at work, to work consistently at high quality, to be a hard worker, to be punctual in coming to work, to spend time on work-related activities, and to be rarely absent from work.

### Statistical analysis

To conduct the analysis, data from all three survey rounds were pooled into a single dataset. For the indices described above, we generated (a) a percent distribution of health workers by whether the health worker was classified as having a motivation score of less than 30, 30–70, or 71–100% of the maximum value of the index and (b) the mean of the index.

To investigate changes in motivational determinants and consequences over time, chi-square statistics were computed. To assess bivariate associations among motivational determinants and motivational consequences, Spearman’s rank order correlation analysis was carried out. In addition, to assess the relationships between motivational determinants and the two motivational outcomes—general job satisfaction and self-perceived conscientiousness—after controlling for other factors, multivariate regression models were estimated. General job satisfaction was estimated using a probit model with the dependent variable being a dichotomous indicator equal to 1 if the respondent reported being either satisfied or very satisfied with his or her job and 0 otherwise. Self-perceived conscientiousness, measured as a continuous index, was estimated through ordinary least squares.

In addition to the motivational measures described above, several other independent variables were included in the pooled cross-sectional regression models. These include the following: sex, which was coded as 1 if the respondent was male and 0 otherwise; type of facility, which was measured as a binary indicator and coded as 1 if the respondent worked in a health center and 0 if the respondent worked in a health clinic or health post; type of health worker, which was a dichotomous variable coded as 1 if the respondent was a nurse and 0 if the respondent was another type of health worker; and years of experience, which is a self-reported continuous indicator. In addition, a set of year dummy variables (2006 and 2009) was included to investigate how job satisfaction and conscientiousness changed over time in ways not explained by other observed variables in the models. Data were analyzed using the statistical software package Stata (Version 13.1).

### Ethical considerations

The research protocol for the study was reviewed and approved by the Ethiopia Health and Nutrition Research Institute Review Board at the Federal Democratic Republic of Ethiopia Ministry of Science and Technology and the Biomedical Institutional Review Board of Tulane University.

## Results

### Characteristics of respondents

Table 1 presents characteristics of respondents by survey year. The percentage of respondents in 2003/4 who were female was about 50%, and this percentage increased in the two subsequent rounds. Very few government facilities employed doctors—public facilities were predominantly staffed by nurses, midwives, and others types of staff. A high percentage of health workers reported working at the facility for less than 2 years. It should be noted that two cadres—health extension workers and health information statisticians and data entry clerks—were introduced in government health facilities between 2003/04 and 2009 as part of the government’s human resources for health strategy. Questions on health extension workers were asked only in the last survey round, as these health workers only began to be deployed in 2006.

**Table 1:** **Percent distribution of sample public sector health workers by selected characteristics and by survey year**

**Indicator**	**2003/4**	**2006**	**2009**
	***N*** **= 234 (%)**	***N*** **= 246 (%)**	***N*** **= 312 (%)**
Sex			
Female	50.4	53.3	60.9
Male	49.6	46.8	39.1
Position			
Doctors	3.4	3.3	0.6
Health officers	1.7	1.6	9.6
Nurses, midwives	44.9	45.5	56.7
Other	50.0	49.6	33.0
Years worked in facility			
Less than 2 years	45.7	40.7	36.5
2 to 5 years	26.5	30.5	34.9
6 or more years	27.8	28.9	28.5

Health workers classified as “other” include health extension workers, laboratory technicians, counselors, pharmacists, and administrative staff

### Self-reported workloads

An analysis of self-reported time use during the 1-month period prior to the surveys suggests that service workloads for health care providers increased during the study period. Table 2 shows the average number of hours respondents reported to have worked per month by type of health service. The results indicate that the average number of hours worked increased for almost all types of services provided. There was a particularly large increase in the hours worked on HIV/AIDS testing, which increased almost threefold between 2003/04 and 2009. The hours allocated for malaria prevention and care decreased between 2003/04 and 2009, a finding that may be attributable to the overall decrease in malaria prevalence in Ethiopia during the study period [15]. Overall, the average number of hours worked per month increased by 6.3 h between 2003/04 and 2009. It should be stressed that the results are self-reported over a relatively long recall period—1 month. Because there may be substantial differences between self-reported and actual time use, the results are only presented in order to present a rough indication of changes in time utilization over the study period. As such, the results should be treated with caution.

**Table 2:** **Percentage of sample public sector health workers who report providing health services, and average reported number of hours worked, by type of service**

**Health services provided**	**% of health workers who provided service in 2003/04 (** ***N*** **= 118)**	**Average hours worked per month**	**% of health workers who provided service in 2006 (** ***N*** **= 125)**	**Average hours worked per month**	**% of health workers who provide service in 2009 (** ***N*** **= 217)**	**Average hours worked per month**	**Change in percentage of health workers who provide services, 2003/04–2009**	**Change in average hours worked per month, 2003/04–2009**
Child health	52.5	62.4	64.0	69.1	35.5	66.6	−16.7***	4.2
Maternal health	50.0	55.8	66.4	63.7	37.3	61.8	−12.7**	6.0
FP	38.1	45.9	48.8	37.7	25.3	60.8	−12.8**	14.9***
HIV/AIDS counseling	12.7	46.2	23.2	46.0	31.3	50.5	18.6***	4.3
HIV/AIDS testing	2.5	14.3	1.6	24.0	22.1	37.8	19.6***	23.5***
STI counseling	10.2	9.3	19.2	15.8	10.1	15.0	−0.1	5.7
STI testing	5.1	5.0	11.2	25.2	6.9	11.2	1.8	6.2
TB care	22.0	62.8	27.2	43.6	16.6	52.8	−5.4	−10.0
Malaria prevention and care	12.7	28.4	20.0	18.2	9.2	15.7	−3.5	−12.7
General outpatient services	45.8	80.8	64.8	78.3	38.7	95.1	−7.1	14.3
General inpatient services	12.7	36.9	18.4	35.7	8.3	38.2	−4.4	1.3
Outreach services	23.7	26.8	8.0	35.6	4.6	40.9	−19.1***	14.1***
Reporting for HIS	-	-	-	-	46.1	4.4	NA	
Other services	42.4	66.7	28.0	67.7	35.5	110.6	−6.9	43.9***
Average number of hours worked	-	177.8	-	208.5	-	184.1	-	6.3

Respondents included in this analysis were restricted to physicians, staff nurses, qualified nurses, qualified midwives, junior nurses, trained birth attendants, and counselors. Results based on sample of workers who report providing service

*P value ≤0.10; **P value of ≤0.05; ***P value of ≤0.01

### Supervision

Some aspects of supervision of government health workers appear to have improved during the study period. As indicated in Table 3, the percentage of health workers who report receiving feedback from their supervisors (either on- or off-site) increased from 67.5% in 2003/4 to 94.2% in 2009, and for those workers whose supervisor was based off-site, the mean number of visits from the supervisor during the 6-month period before the survey increased from 1.9 to 4.4 visits.

**Table 3:** **Percentage of sample public sector health workers reporting to have been supervised**

**Supervision**	**2003/04**	**2006**	**2009**	**Change, 2003/04–2009**
	**(** ***N*** **= 234)**	**(** ***N*** **= 246)**	**(** ***N*** **= 312)**	
Percentage of health workers who report that their supervisor is based on-site	51.3%	61.4%	78.5%	27.2***
Mean number of times health worker supervised by off-facility supervisor was visited during the last 6 months	1.86	3.01	4.15	2.29***
Percentage of health workers who received feedback from their supervisor	67.5%	94.3%	94.2%	26.7***

*P value ≤0.10; **P value of ≤0.05; ***P value of ≤0.01

### Univariate analysis: determinants of motivation

Table 4 presents descriptive results for the four indices of the determinants of health worker motivation assessed in the study—resource availability, financial rewards, pride, and self-efficacy.

**Table 4:** **Indices of the determinants health worker motivation among sample public sector health workers**

**Motivation index**	**Percentage of providers in 2003/04**	**Percentage of providers in 2006**	**Percentage of providers in 2009**	**Change in percentage points, 2003/04–2009**
	**(** ***N*** **= 234)**	**(** ***N*** **= 246)**	**(** ***N*** **= 312)**	
Resource availability				
30% and below	85.5	85.0	84.6	−0.9
31–70%	12.0	13.4	12.5	0.5
71–100%	2.6	1.6	2.9	0.3
Average index	20.4%	10.9%	11.3%	−9.1***
Financial rewards				
30% and below	97.0	96.7	95.2	−1.8
31–70%	3.0	3.2	4.2	1.2
71–100%	0.0	0.0	0.6	0.6
Average index	10.9%	9.5%	7.3%	−3.6***
Pride				
30% and below	50.4	37.0	30.4	−20.0
31–70%	33.3	47.6	48.1	14.8
71–100%	16.2	15.5	21.6	5.4
Average index	36.2%	42.9%	47.5%	11.3***
Self-efficacy				
30% and below	31.6	32.1	9.9	−21.7
31–70%	59.4	60.6	63.8	4.4
71–100%	9.0	7.3	26.4	17.4
Average index	46.2%	46.8%	61.1%	14.9***

*P value ≤0.10; **P value of ≤0.05; ***P value of ≤0.01

#### Resource availability

The resource availability index measures health workers’ perceptions of the availability of equipment, supplies, and other resources in their health facility. The index value is very low for all 3 years, indicating that workers largely disagreed with statements regarding the availability of equipment and supplies. Moreover, there was a decrease in the overall index over the study period. In 2009, the average index of health workers’ satisfaction with resources available was 11.3% points, a 9.1% decrease from 2003/04 (*P* value <0.001).

#### Financial rewards

The level of the index measuring health workers’ satisfaction with their remuneration is low in all three rounds, indicating high levels of dissatisfaction. As Table 6 shows, the average index level was 7.3% in 2009, a 3% decrease from 2003/04 (*P* value <0.001).

#### Pride

Despite a statistically significant increase in the pride index from 2003/04 to 2009, the average was only 47.5% among government health workers in 2009, an increase of 11.3% from the 2003/04 level. The pride index is higher among health providers working in health centers than among those working in clinics/health posts (results not reported).

#### Self-efficacy

Self-efficacy measures the extent to which health workers believe they can be successful in their work. The survey results suggest that self-efficacy increased among the public providers from 46.2% in 2003/04 to 61.1% in 2009, a change of 14.9 percentage points (*P* < 0.01). The improvement in self-efficacy occurred across all types of health facilities—health centers, posts, and clinics—though the increase was slightly higher among workers in clinics and health posts (results not reported). This differential could be attributed to the contributions of HEP and the recognition that the HEWs have received.

### Univariate analysis: motivational outcomes

#### Overall job satisfaction

Job satisfaction has previously been defined as a worker’s perspective of how well his/her job provides those things that he/she views as important (Locke and Latham 1976). A health worker’s job satisfaction is thought to be important because of its hypothesized association with internal motivation and, as a result, overall job performance. Table 5 indicates that 79.5% of workers in the sample were overall satisfied or very satisfied with their job, a 23.9% increase from 2004. This change over the study period and the changes in the other indicators of job satisfaction reported in Table 5 are statistically significant (*P* value ≤0.01).

**Table 5:** **Job satisfaction among sample public sector health workers**

**Job satisfaction**	**Percentage of providers in 2003/04**	**Percentage of providers in 2006**	**Percentage of providers in 2009**	**Change in percentage of providers, 2003/04–2009**
	**(** ***N*** **= 234) (%)**	**(** ***N*** **= 246) (%)**	**(** ***N*** **= 312) (%)**	
Overall job satisfaction	55.6	80.1	79.5	23.9***
Overall satisfaction with chance to accomplish something worthwhile	59.4	61.8	76.6	17.2***
Satisfaction with the chance to learn something new	21.8	20.8	35.9	14.1***
Overall satisfaction with the chance to do something that makes you feel good as a person	40.6	55.3	58.7	18.1***

*P value ≤0.10; **P value of ≤0.05; ***P value of ≤0.01

#### Self-perceived conscientiousness

The index on self-perceived conscientiousness reflects workers’ assessment of their reliability as health workers. As indicated in Table 6, the index values are high in all three rounds. In 2009, the average index increased slightly among public sector health workers by 5.5 percentage points (*P* < 0.01).

**Table 6:** **Index of self-perceived conscientiousness among sample public sector health workers**

**Index**	**Percentage of providers in 2003/04**	**Percentage of providers in 2006**	**Percentage of providers in 2009**	**Change in percentage, 2003/04–2009**
	**(** ***N*** **= 234)**	**(** ***N*** **= 246)**	**(** ***N*** **= 312)**	
30% and below	3.4	2.0	1.3	−2.1
31–70%	38.0	52.0	35.3	−2.7
71–100%	58.5	45.9	63.4	4.9
Average index	72.3%	68.2%	77.8%	5.5***

*P value ≤0.10; **P value of ≤0.05; ***P value of ≤0.01

### Bivariate analysis

Table 7 presents pairwise correlations between the indicators of the determinants of motivation, overall job satisfaction, and satisfaction with financial rewards. As can be seen from Table 9, each of the coefficients for the pairs of motivational determinants is positive and statistically significant (*P* < 0.05), with the exception of the coefficients of (a) financial rewards and pride and (b) financial rewards and self-efficacy. With respect to the correlations between motivational determinants and consequences, pride and self-efficacy are positively and significantly associated with overall job satisfaction, and resource availability, self-efficacy, and satisfaction with financial rewards were positively and significantly associated with conscientiousness.

**Table 7:** **Spearman’s rank order correlation coefficients for indicators of the determinants and consequences of worker motivation among sample public sector health workers**

**Index/indicator**	**Resource availability**	**Pride**	**Self-efficacy**	**Satisfaction with financial rewards**	**Overall job satisfaction**
Determinants					
Resource availability	1.0000				
Pride	0.0765*	1.0000			
Self-efficacy	0.0828*	0.3780*	1.0000		
Satisfaction with financial rewards	0.1234*	0.0152	−0.0446	1.000	
Consequences					
Overall job satisfaction	−0.0013	0.3343*	0.2812*	0.0398	1.0000
Conscientiousness	0.1025*	0.3233	0.5573*	−0.1042*	0.2119*

*P value of ≤0.05

### Multivariate analysis

Table 8 presents multivariate results of the determinants of general satisfaction and self-perceived conscientiousness. As shown in the table, several motivational determinants were found to be significantly associated with the dependent variables, after controlling for the other variables. Pride and self-efficacy are positively and significantly associated with both general satisfaction and conscientiousness, while resource availability and financial rewards are positively and significantly associated with general satisfaction, but not conscientiousness. In terms of the other independent variables in the model, being a woman and working for a health center, as opposed to a health post or health clinic, emerged as statistically significant predictors of general job satisfaction, but not self-perceived conscientiousness. To explore the robustness of our findings, we also estimated the models based on a restricted sample of doctors, nurses, and midwives. The results were very similar to those based on the full sample with the following exceptions: in the model of general satisfaction, resource availability was insignificant, and in the model of conscientiousness, the indicator of the chance to accomplish something worthwhile was insignificant (results not reported).

**Table 8:** **Multivariate regression results of the determinants of general job satisfaction and self-perceived conscientiousness among sample public sector health workers**

**Independent variable**	**Model**			
	**Probit model of general job satisfaction**		**OLS model of conscientiousness**	
	**Coefficient**	**Standard error**	**Coefficient**	**Standard error**
Survey year (reference: 2003/4)				
2006	0.917***	0.148	−5.055***	1.667
2009	0.473***	0.145	−3.841**	1.693
Demographic and worker characteristics				
Health center (reference: health clinics and health posts)	0.343***	0.131	1.333	1.317
Nurse (reference: other workers)	0.066	0.115	−1.006	1.317
Years of experience	0.011	0.009	0.031	0.098
Female (reference = males)	0.370***	0.117	0.031	1.344
Individual differences				
Self-efficacy	0.011***	0.003	0.501***	0.035
Perceived contextual variables				
Resource availability	0.006*	0.004	0.041	0.035
Pride	0.012***	0.002	0.120***	0.026
Financial rewards	0.013*	0.006	−0.126**	0.055
Chance to accomplish something worthwhile	0.922***	0.114	2.152	0.410
Constant	−2.129***	0.236	42.559***	2.433
Pseudo R-square (probit) or R-square (OLS)	0.35		0.27	
*N*	792		792	

*P value ≤0.10; **P value of ≤0.05; ***P value of ≤0.01

## Discussion

Although Ethiopia has experienced significant improvements in health outcomes in recent years, there continue to be substantial problems in the stock, distribution, and performance of health workers. According to a recent assessment of the human resources for health situation in the country, there are a number of indications that the overall performance of health workers is negatively impacted by low levels of health worker motivation and job satisfaction, particularly in the areas of salary, access to further training and promotion, lack of mentoring, and inadequate physical conditions in the workplace [16]. These issues can potentially lead to several performance-related problems that are inter-connected, including the inefficient delivery of health care services, non-responsiveness to patient needs, and absenteeism.

The purpose of the study is to empirically investigate changes in job satisfaction and potential determinants and consequences of motivation among public sector primary health care workers in Ethiopia over three points in time—2003/04, 2006, and 2009. During the study period, Ethiopia introduced several initiatives in order to improve the availability and quality of health services, including the Health Extension Program and district-based planning. Moreover, there was a rapid scale-up of HIV/AIDS, tuberculosis, and malaria services during the period that was made possible with the support of global health initiatives such as GFATM and PEPFAR.

The results suggest that overall job satisfaction and self-perceived conscientiousness, considered in the study to be two consequences of motivation, significantly increased during the study period and were relatively high by the time of the last survey. For example, in 2009, 80% of respondents agreed with the statement that, overall, they were satisfied with their job—and the percentile score for the index of self-perceived conscientiousness was 78. What factors contributed to these improved outcomes? The findings from the multivariate analysis suggests that increases in self-efficacy and pride played important roles, as they were positively and significantly associated with both outcomes, after controlling for other factors, while the “chance to accomplish something worthwhile” was found to have a positive effect on general job satisfaction, but not self-perceived conscientiousness. Improvements in health worker supervision may have also played a role, as suggested by the substantial increases over the study period in the proportion of workers who reported receiving feedback from their supervisor.

Although general job satisfaction improved, respondents expressed substantial frustrations with their job. For example, perceived resource availability and satisfaction with financial rewards were found to be very low in all survey rounds, which is consistent with the findings of Serra et al. [6] and Lindelow and Serneels [7]. Moreover, both measures actually worsened over the study period. Interestingly, the finding regarding perceived resource availability is at odds with results of the facility survey carried out in the same primary health care facilities, which suggest that the actual availability of equipment, laboratory supplies, and infrastructure increased over the 2003/4 to 2009 study period [8]. The reasons for the discrepancy between perceived and actual availability of facility resources are unclear, but changes in workers’ expectations of what resources *should* be available may be part of the explanation.

There are a number of limitations to this analysis. First, the sample of facilities was not selected randomly. As such, the sample is not generalizable of the four regions included in the study. Second, our dataset did not include information on many other potential determinants of health worker motivation—such as worker values, work ethics, job preferences, and worker expectations. Nor did our dataset include good measures of the actual performance and job tenure of health workers, which prevented us from investigating how the motivational determinants influence health worker performance and turnover. Third, the sample was restricted to health workers at primary health care facilities, which prevented us from investigating health worker motivation among physicians and other types of cadres working in hospitals. Finally, our sample size was not large enough to disaggregate the analysis by type of primary health worker. As such, the study findings may mask important differences that exist between health workers.

Despite these limitations, the study contributes to the health worker motivation literature in several ways. First, this is the first quantitative study of health worker motivation in Ethiopia that incorporates multiple dimensions of motivational determinants and consequences. The findings suggest that, in the Ethiopian context, a myriad of factors influence health worker motivation, including satisfaction with financial rewards, opportunities for development, resource availability, and perceptions of organizational culture. These results are consistent with previous studies of health worker motivation in other settings in sub-Saharan Africa and elsewhere [2-4,9,12,13,17,18]. Secondly, the availability of three rounds of survey data collected with the same survey instrument in the same facilities provided us with a unique opportunity to assess changes in motivational determinants and consequences over time. The finding that job satisfaction and conscientiousness improved over the study despite most workers reporting high levels of dissatisfaction with financial rewards provides new temporal evidence pointing to the importance of non-financial factors, as most previous quantitative studies on health worker motivation are based on cross-sectional data only [2-4,9,12,13,17,18].

## Conclusions

Overall, the findings of the study suggest that, in the Ethiopian context, several factors thought to be associated with motivation of public sector health workers increased over the 2003/4 to 2009 study period but that perceived resource availability and satisfaction with financial rewards remained very low. Although the findings do not point to specific interventions that should be introduced, they do highlight the complexity of health worker motivation and support the premise that both financial and non-financial factors are important determinants of job satisfaction and health worker motivation.

## Endnotes

^1^Information on workloads were gathered by asking respondents “how many hours on average per month do you provide _____ service?”. The question was asked for each service category listed in Table 2.

## Additional file

Additional file 1: Table S1. Cronbach’s Alpha of motivation indices by survey round. Cronbach’s alpha was used to examine the inter-reliability of the scales used and most of the scales had acceptable alpha levels.
