# Supplementary material for: Job satisfaction and motivation among public sector health workers: evidence from Ethiopia
Source: Hum Resour Health. 2015 Oct 29;13:83. doi: 10.1186/s12960-015-0083-6 (PMC4625466; doi:10.1186/s12960-015-0083-6)
Supplement: Additional file 1: Table S1. — Cronbach’s Alpha of motivation indices by survey round. Cronbach’s alpha was used to examine the inter-reliability of the scales used and most of the scales had acceptable alpha levels. [file 12960_2015_83_MOESM1_ESM.doc]

**Additional file 1: Table S1. Cronbach’s Alpha of motivation indices by survey round.**

| **Motivation Indices** | **Cronbach’s Alpha** | | | |
| --- | --- | --- | --- | --- |
|  | **2003/4** | **2006** | **2009** | **Total pooled sample** |
| Pride Index | 0.870 | 0.781 | 0.734 | 0.798 |
| Satisfaction with financial rewards | 0.777 | 0.760 | 0.825 | 0.794 |
| Self-Efficacy | 0.681 | 0.604 | 0.595 | 0.641 |
| Resource Availability | 0.961 | 0.861 | 0.910 | 0.749 |
| Conscientiousness | 0.802 | 0.860 | 0.825 | 0.832 |
| All motivation variables (20) | 0.845 | 0.868 | 0.842 | 0.838 |
